# Elevated plasma D-dimer levels are associated with the poor prognosis of critically ill children

**DOI:** 10.3389/fped.2022.1001893

**Published:** 2022-09-23

**Authors:** Guan Wang, Junhui Liu, Rui Xu, Xinjie Liu

**Affiliations:** ^1^Department of Pediatrics, Qilu Hospital of Shandong University, Jinan, China; ^2^Qilu Hospital of Shandong University, Jinan, China

**Keywords:** plasma D-dimer, coagulation dysfunction, critically ill children, in-hospital mortality, pediatric intensive care unit (PICU)

## Abstract

**Background:**

D-dimer has been shown as a valuable predictor for the prognosis of sepsis. But the prognostic association of an elevated D-dimer with adverse outcomes of all critical illnesses in pediatric intensive care unit (PICU) has received far less emphasis.

**Methods:**

This was a single-center retrospective study, including 7,648 critical patients aged between 28 days and 18 years from the pediatric intensive care (PIC) database from 2010 to 2018. The primary outcome was the in-hospital mortality rate.

**Results:**

Higher levels of D-dimer, INR, PT, APTT, and lower Fib were observed in the non-survivor group (all *P* < 0.001). D-dimer, INR, PT and APTT were independent risk factors for prognosis in critically ill children. There was the highest AUROC in D-dimer for predicting in-hospital mortality of critically ill patients compared with INR, PT, APTT, and Fib (D-dimer: 0.77 vs. INR: 0.73 vs. PT: 0.73 vs. APTT: 0.64 vs. Fib: 0.60). The cut-off value, sensitivity, and specificity of D-dimer were 1.53, 0.65, and 0.77, respectively. Subgroup analysis showed a stable evaluation effectiveness of D-dimer for predicting in-hospital mortality of critically ill patients in the age and gender groups.

**Conclusions:**

We found poorer coagulation function in the non-survivors compared with the survivors. Among the coagulation indicators, D-dimer was most strongly associated with in-hospital mortality of unselected critically ill children.

## Introduction

D-dimer has been shown to be a potential predictor for the poor prognosis of sepsis in both adult and pediatric patients (1–3). But the prognostic association of an elevated D-dimer with adverse outcomes of all critical illnesses in pediatric intensive care unit (PICU) has received far less emphasis.

As a soluble fibrin degradation product, D-dimer comes from the orderly breakdown of thrombi by the fibrinolytic system (4). Numerous studies have shown that D-dimer was a valuable marker in predicting the outcome of some infectious diseases. One study indicated that pediatric patients with septic shock had a higher level of D-dimer in the non-survivor group (5). High levels of D-dimer have a reported association with 28-day mortality in adult patients with infection or sepsis identified in the emergency department (1). Recently, one study about coronavirus disease 2019 (COVID-19) found that D-dimer >1 μg/mL was relative to the fatal outcome (6). The effect mechanism of D-dimer includes leading to a systemic pro-inflammatory cytokine response, which is a mediator of atherosclerosis, directly promoting plaque rupture through local inflammation, inducing pro-coagulant factors and hemodynamic changes that predispose to ischemia, and thrombosis (7–9).

It is crucial for clinicians to identify predictive biomarkers in unselected critically ill children for early detection and timely treatment, which may significantly reduce the death rate of patients. Critical illnesses in PICU mainly include infectious diseases, renal and heart failure, severe trauma, intracranial bleeding, severe connective tissue disease, and other conditions (10). In addition to the findings described above, D-dimer has also been proven relative to the prognosis of other diseases, including closed brain injury and systemic lupus erythematosus (SLE) (11, 12). Dysregulation of coagulation homeostasis may be associated with the endothelial injury of these diseases (13–15), which results in the formation of microthrombi to occlude vessels and leads to multiple organ failures eventually (16). It follows that D-dimer is involved in the pathological process and is strongly associated with the poor prognosis in critical illnesses. However, to date, no studies have explored the association between D-dimer and overall in-hospital mortality of critical illnesses in PICU.

In the present study, we retrospectively collected and analyzed the D-dimer levels in patients admitted to PICU in a large sample size, aiming to investigate the prognostic performance of D-dimer in predicting overall in-hospital mortality in unselected critically ill children.

## Methods

### Study participants

**Figure 1:**
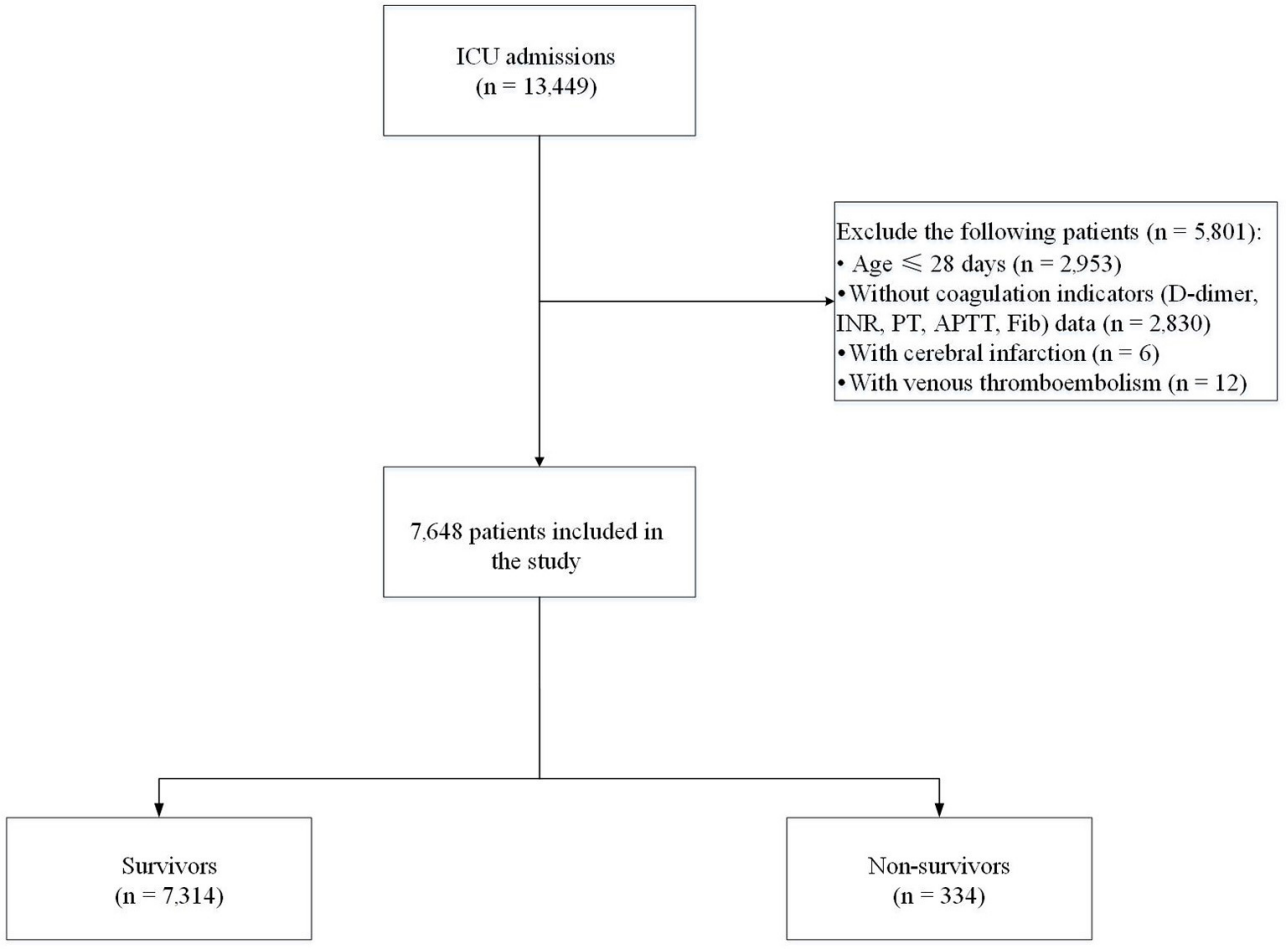
Flow chart of recruitment of the study population. ICU, intensive care unit; INR, international normalized ratio; PT, prothrombin time; APTT, activated partial thromboplastin time; Fib, fibrinogen.

We collected the clinical data of patients from the pediatric intensive care (PIC) database (version 1.1.0), which contains hospital clinical records from 2010 to 2018 of the Children's Hospital of Zhejiang University School of Medicine (Hangzhou, China) (17). The patient's medical history, standard laboratory values, and vital signs were documented. Individuals aged ≤ 28 days or >18 years, individuals without coagulation indicators including D-dimer, prothrombin time (PT), international normalized ratio (INR), activated partial thromboplastin time (APTT), and fibrinogen (Fib) data, and patients with cerebral infarction or with venous thromboembolism were not included in this study. The final cohort comprised 7,648 patients. Seven thousand three hundred and fourteen patients who survived to hospital discharge were included in the survivor group and 334 patients who died in hospital were included in the non-survivor group (Figure 1). The Institutional Review Board of the Children's Hospital of Zhejiang University School of Medicine (Hangzhou, China) approved the survey protocol. The requirement for individual patient consent was waived because the study did not impact clinical care, and all protected health information was de-identified.

### Data collection

The clinical data collected mainly included patient characteristics, such as age, gender, and ICU days, and laboratory values, such as coagulation indicators. All laboratory variables were obtained from the first blood draw after PICU admission. The diagnosis was based on the International Statistical Classification of Diseases and Related Health Problems, 10th edition (ICD-10) system. The primary outcome was in-hospital mortality.

### Statistical analysis

Continuous variables were expressed as mean ± standard deviation (SD) and categorical variables were described as *n* (%). Continuous variables were analyzed using *t*-test to examine the differences in clinical characteristics and laboratory data in the study population, and categorical variables using chi-square analysis. Multivariable logistic regression models were developed to adjust for potential confounders in the association between coagulation indicators and in-hospital mortality (primary outcome), shown as odds ratios (ORs) and 95% confidence intervals (CIs). Model I was a non-adjusted model, while Model II was adjusted for age, sex, bacteremia, vasopressors use, white blood cell (WBC), hemoglobin, platelet (PLT), alanine transaminase (ALT), albumin, creatinine, lactate, C reactive protein (CRP) and principal diagnosis on ICU admission. In addition, restricted cubic spline (RCS) was used to evaluate the linear or non-linear relations between coagulation parameters and in-hospital mortality. Subgroup analyses were performed to examine whether the association between coagulation indicators (D-dimer and INR) and in-hospital mortality differed across subgroups classified by age, gender, bacteremia, and primary diagnosis. Receiver operating characteristic (ROC) analysis was performed and the area under the curve (AUC) was calculated to assess the predictive value of coagulation indicators in children with a critical illness. The Youden Index was calculated to determine the best cut-off value for predicting in-hospital mortality. All statistical analyses were performed using R version 3.4.3. (https://www.r-project.org, The R Foundation for Statistical Computing, Vienna, Austria) and EmpowerStats (http://www.empowerstats.com, X&Y Solutions, Inc., Boston, Mass, USA) software packages. Statistical significance was defined as a two-sided *P*-value of <0.05.

## Results

### Clinical and laboratory characteristics of the study population

Table 1 showed the clinical and laboratory characteristics of the survivors and non-survivors. There was no significant difference in age between the two groups. Longer ICU days (14.85 ± 17.56 vs. 5.51 ± 15.85, *P* < 0.001) and a higher rate of vasopressors use (47.90 vs. 35.90%, *P* < 0.001) were shown in the non-survivor group due to their illness was more severe than the survivor group. Respiratory diseases and cardiovascular diseases were the most common type of illness in the non-survivor group (23.35%) and the survivor group (28.38%), respectively. Bacteremia was more common in the non-survivor group (35.33%, *P* < 0.001). All laboratory variables were obtained from the first blood draw after PICU admission, within 42 h from admission. There were higher levels of WBC, CRP, ALT, and lactate and lower levels of albumin, PLT, and hemoglobin in the non-survivor group (all *P* < 0.001), indicating more severe inflammation, liver dysfunction, poor circulation, and anemia in the non-survivor group. Most importantly, our findings showed a significant difference in the coagulation parameters, including D-dimer, INR, PT, APTT, and Fib between the two groups. Higher D-dimer (5.22 ± 6.56 vs. 1.68 ± 3.78), INR (1.40 ± 0.88 vs. 1.07 ± 0.44), PT (16.80 ± 10.65 vs. 12.78 ± 5.27), APTT (44.82 ± 25.62 vs. 34.09 ± 12.88), and lower Fib (2.00 ± 1.28 vs. 2.24 ± 1.02) were observed in the non-survivor group compared with the survivor group (all *P* < 0.001), suggesting poorer coagulant function in the non-survivor group, which indicated that coagulation parameters may play an important role in predicting the prognosis of critically ill patients in PICU.

**Table 1:** Baseline clinical and laboratory characteristics of the study population.

**Characteristics**	**All**	**Survivors**	**Non-survivors**	***P*** **-value**
Number	7,648	7,314	334	
Age (months)	39.77 ± 46.31	39.68 ± 46.11	41.94 ± 50.49	0.382
Males (%)	4311 (56.37%)	4097 (56.02%)	214 (64.07%)	0.004
Hospital days	16.19 ± 17.95	16.20 ± 17.92	15.98 ± 18.61	< 0.001
ICU days	5.92 ± 16.04	5.51 ± 15.85	14.85 ± 17.56	< 0.001
Principal diagnosis on ICU admission, *n* (%)				< 0.001
Congenital disease	1,100 (14.38%)	1,085 (14.83%)	15 (4.49%)	
Hematological disease	311 (4.07%)	268 (3.66%)	43 (12.87%)	
Cardiovascular disease	2,129 (27.84%)	2,076 (28.38%)	53 (15.87%)	
Neurologic disease	731 (9.56%)	683 (9.34%)	48 (14.37%)	
Digestive disease	640 (8.37%)	623 (8.52%)	17 (5.09%)	
Neoplasm	733 (9.58%)	712 (9.73%)	21 (6.29%)	
Respiratory disease	744 (9.73%)	666 (9.11%)	78 (23.35%)	
Trauma	373 (4.88%)	344 (4.70%)	29 (8.68%)	
Others	887 (11.60%)	857 (11.72%)	30 (8.98%)	
Vasopressors, *n* (%)	2,786 (36.43%)	2,626 (35.90%)	160 (47.90%)	< 0.001
Bacteremia, *n* (%)	1,382 (18.07%)	1,264 (17.28%)	118 (35.33%)	< 0.001
**Coagulation parameters**				
D-dimer (mg/L)	1.84 ± 4.01	1.68 ± 3.78	5.22 ± 6.56	< 0.001
INR	1.08 ± 0.47	1.07 ± 0.44	1.40 ± 0.88	< 0.001
PT (s)	12.95 ± 5.67	12.78 ± 5.27	16.80 ± 10.65	< 0.001
APTT (s)	34.56 ± 13.85	34.09 ± 12.88	44.82 ± 25.62	< 0.001
Fib (g/L)	2.23 ± 1.03	2.24 ± 1.02	2.00 ± 1.28	< 0.001
**Others laboratory parameters**				
Albumin (g/L)	41.18 ± 6.25	41.41 ± 6.07	36.08 ± 7.78	< 0.001
ALT (U/L)	65.03 ± 351.58	60.83 ± 322.70	157.49 ± 738.20	< 0.001
Creatinine (umol/L)	71.49 ± 428.29	72.17 ± 437.71	56.50 ± 60.09	0.039
CRP (mg/L)	22.43 ± 32.97	22.02 ± 32.59	30.69 ± 39.21	< 0.001
WBC (× 10^9^ /L)	11.22 ± 24.16	10.98 ± 22.63	16.34 ± 46.20	< 0.001
PLT (× 10^9^ /L)	324.38 ± 145.92	328.02 ± 143.29	244.76 ± 177.18	< 0.001
Hemoglobin (g/L)	113.76 ± 20.70	114.13 ± 20.36	105.69 ± 25.83	< 0.001
Lactate (mmol/L)	2.08 ± 1.86	2.00 ± 1.66	3.99 ± 3.84	< 0.001

ICU, intensive care unit; INR, international normalized ratio; PT, prothrombin time; APTT, activated partial thromboplastin time; Fib, fibrinogen; ALT, alanine transaminase; CRP, C reactive protein; WBC, white blood cell; PLT, platelet.

### The association between coagulation parameters and in-hospital mortality of critically ill children

The relationship between coagulation parameters and in-hospital mortality of pediatric critically ill patients was analyzed by different models of multivariable logistic regression (Table 2). We found that D-dimer (OR 1.05, 95% CI 1.03–1.07, *P* < 0.001), INR (OR 1.24, 95% CI 1.03–1.48, *P* = 0.020), PT (OR 1.02, 95% CI 1.00–1.03, *P* = 0.026), APTT (OR 1.01, 95% CI 1.00–1.02, *P* = 0.001), and Fib (OR 0.81, 95% CI 0.71–0.91, *P* < 0.001) were significantly associated with the in-hospital mortality of critically ill children after adjusting for age, sex, bacteremia, vasopressors use, WBC, hemoglobin, PLT, ALT, albumin, creatinine, lactate, CRP and principal diagnosis on ICU admission, indicating that D-dimer, INR, PT and APTT were independent risk factors and Fib was an independent protective factor for prognosis in critically ill children. RCS was used to test the non-linear relation between coagulation parameters and in-hospital mortality (Supplementary Figure 1). The results showed that the coagulation parameters and in-hospital mortality were all non-linear relations (all *P* for non-linear trend <0.001) and we converted D-dimer, INR, PT, APTT and Fib to categorical variables according to quartiles. After stratifying D-dimer to quartiles, the first quartile group (0.01–0.21) was used as a reference. D-dimer level in the third quartile group (0.46–1.51 for D-dimer, OR 2.20, 95% CI 1.27–3.82, *P* = 0.005) and the fourth quartile group (1.51–56.86 for D-dimer, OR 4.86, 95% CI 2.84–8.31, *P* < 0.001) were both significantly related to raised risks of in-hospital mortality of the patients, and a higher D-dimer value was associated with a higher risk of in-hospital mortality. The similar trend was also seen in PT. Only extremely high group of INR (the fourth quartile group, 1.07–17.38) was related to raised risks of in-hospital mortality of the patients (OR 3.30, 95% CI 2.10–5.19, *P* < 0.001). Interestingly, there was a U-shaped relationship between APTT and in-hospital mortality. The second quartile group (27.8–31.3) and the third quartile group (31.3–36.7) of APTT yielded lower in-hospital mortality in contrast to patients with APTT value of 15.3–27.8 and 36.7–190.2 (*P* < 0.05 for both). As a protective factor, Fib levels in the second to fourth quartile groups were all related to decreased risks of in-hospital mortality of the patients (1.62–2.03: OR 0.49, 95% CI 0.34–0.72, *P* < 0.001; 2.03–2.57: OR 0.45, 95% CI 0.31–0.68, *P* < 0.001; 2.57–28.7: OR 0.56, 95% CI 0.40–0.79, *P* < 0.001).

**Table 2:** Relationship between coagulation parameters and in-hospital mortality in different models of multivariable logistic regression.

	**Model I**			**Model II**		
	**OR**	**95% CI**	***P-*** **value**	**OR**	**95% CI**	***P-*** **value**
D-dimer	1.10	(1.09, 1.12)	<0.001	1.05	(1.03, 1.07)	<0.001
**D-dimer** ^**Quartile**^						
0.01–0.21		1			1
0.21–0.46	1.19	(0.66, 2.16)	0.566	1.13	(0.60, 2.10)	0.712
0.46–1.51	3.68	(2.23, 6.06)	<0.001	2.20	(1.27, 3.82)	0.005
1.51–56.86	12.11	(7.62, 19.23)	<0.001	4.86	(2.84, 8.31)	<0.001
D-dimer-*P* for trend		<0.001			<0.001
INR	1.81	(1.59, 2.07)	<0.001	1.24	(1.03, 1.48)	0.020
**INR** ^**Quartile**^						
0.66–0.93		1			1
0.93–0.98	1.09	(0.66, 1.80)	0.733	1.22	(0.70, 2.11)	0.489
0.98–1.07	1.54	(0.99, 2.40)	0.054	1.62	(1.00, 2.64)	0.051
1.07–17.38	6.46	(4.41, 9.47)	<0.001	3.30	(2.10, 5.19)	<0.001
INR-*P* for trend		<0.001			<0.001
PT	1.05	(1.04, 1.06)	<0.001	1.02	(1.00, 1.03)	0.026
**PT** ^**Quartile**^						
7.8–11.2		1			1
11.2–11.8	1.08	(0.66, 1.75)	0.762	1.30	(0.76, 2.23)	0.339
11.8–12.9	1.71	(1.12, 2.60)	0.013	1.85	(1.15, 2.96)	0.011
12.9–202.8	6.25	(4.32, 9.05)	<0.001	3.16	(2.02, 4.96)	<0.001
PT-*P* for trend		<0.001			<0.001
APTT	1.03	(1.02, 1.03)	<0.001	1.01	(1.00, 1.02)	0.001
**APTT** ^**Quartile**^						
15.3–27.8		1			1
27.8–31.3	0.34	(0.22, 0.53)	<0.001	0.39	(0.24, 0.63)	<0.001
31.3–36.7	0.54	(0.37, 0.79)	0.001	0.66	(0.44, 0.99)	0.046
36.7–190.2	2.45	(1.87, 3.22)	<0.001	1.54	(1.10, 2.16)	0.011
APTT-*P* for trend		<0.001			0.002
Fib	0.75	(0.66, 0.86)	<0.001	0.81	(0.71, 0.91)	<0.001
**Fib** ^**Quartile**^						
0.18–1.62		1			1
1.62–2.03	0.28	(0.20, 0.39)	<0.001	0.49	(0.34, 0.72)	<0.001
2.03–2.57	0.27	(0.19, 0.37)	<0.001	0.45	(0.31, 0.68)	<0.001
2.57–28.7	0.52	(0.40, 0.68)	<0.001	0.56	(0.40, 0.79)	<0.001
Fib-*P* for trend		<0.001			<0.001

OR, odds ratio; CI, confidence interval; INR, international normalized ratio; PT, prothrombin time; APTT, activated partial thromboplastin time; Fib, fibrinogen. Model I: non-adjusted model. Model II: adjusted for age, sex, bacteremia, vasopressors use, WBC, hemoglobin, PLT, ALT, albumin, creatinine, lactate, CRP and principal diagnosis on ICU admission.

### The predictive value of coagulation parameters for in-hospital mortality of critically ill patients

**Figure 2:**
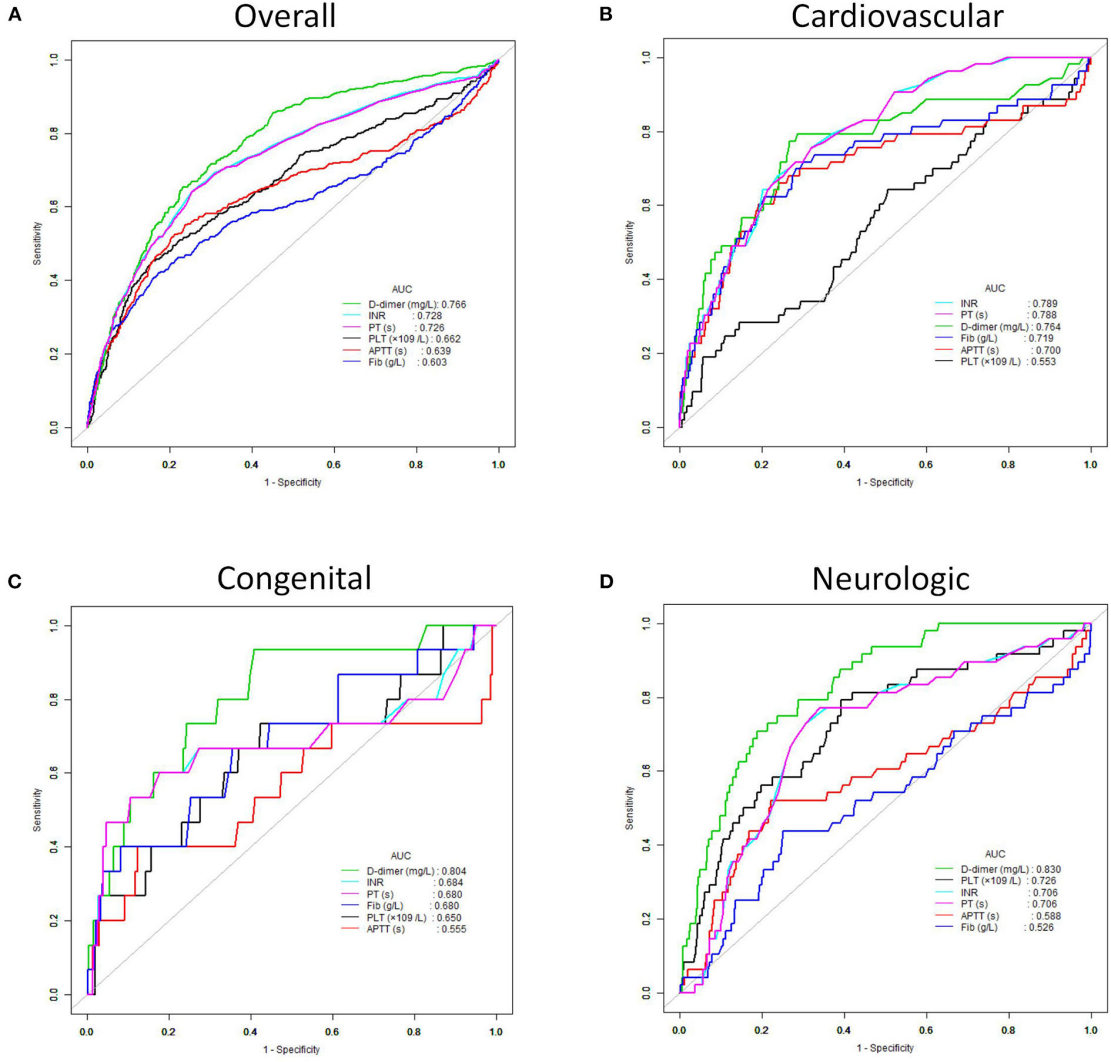
Receiver operating characteristic (ROC) curves of the D-dimer, INR, PT, APTT, Fib and PLT for predicting in-hospital mortality. **(A)** All critically ill patients. **(B)** Patients with cardiovascular disease. **(C)** Patients with congenital disease. **(D)** Patients with neurologic disease.

ROC analysis was performed to assess the performance of coagulation parameters in predicting in-hospital mortality (Table 3). There was the highest AUC in D-dimer in predicting in-hospital mortality of critically ill patients compared with INR, PT, APTT, and Fib (D-dimer: 0.77, 95% CI 0.74–0.79 vs. INR: 0.73, 95% CI 0.70–0.76 vs. PT: 0.73, 95% CI 0.69–0.76 vs. APTT: 0.64, 95% CI 0.60–0.68 vs. Fib: 0.60, 95% CI 0.56–0.64). The cut-off value, sensitivity, and specificity of D-dimer were 1.53, 0.65, and 0.77, respectively. Likewise, in congenital and neurologic diseases, D-dimer also had the best performance in predicting in-hospital mortality of critically ill patients (AUC: congenital 0.804, neurologic 0.830) (Figure 2).

**Table 3:** Performance of variables in predicting in-hospital mortality.

**Variable**	**AUC (95% CI)**	**Cut-off value**	**Sensitivity**	**Specificity**
D-dimer	0.77 (0.74, 0.79)	1.53	0.65	0.77
INR	0.73 (0.70, 0.76)	1.06	0.66	0.73
PT	0.73 (0.69, 0.76)	12.75	0.64	0.75
APTT	0.64 (0.60, 0.68)	36.65	0.55	0.76
Fib	0.60 (0.56, 0.64)	1.48	0.42	0.83

AUC, area under curve; INR, international normalized ratio; PT, prothrombin time; APTT, activated partial thromboplastin time; Fib, fibrinogen.

### Subgroup analysis of the association between D-Dimer, INR and in-hospital mortality

**Figure 3:**
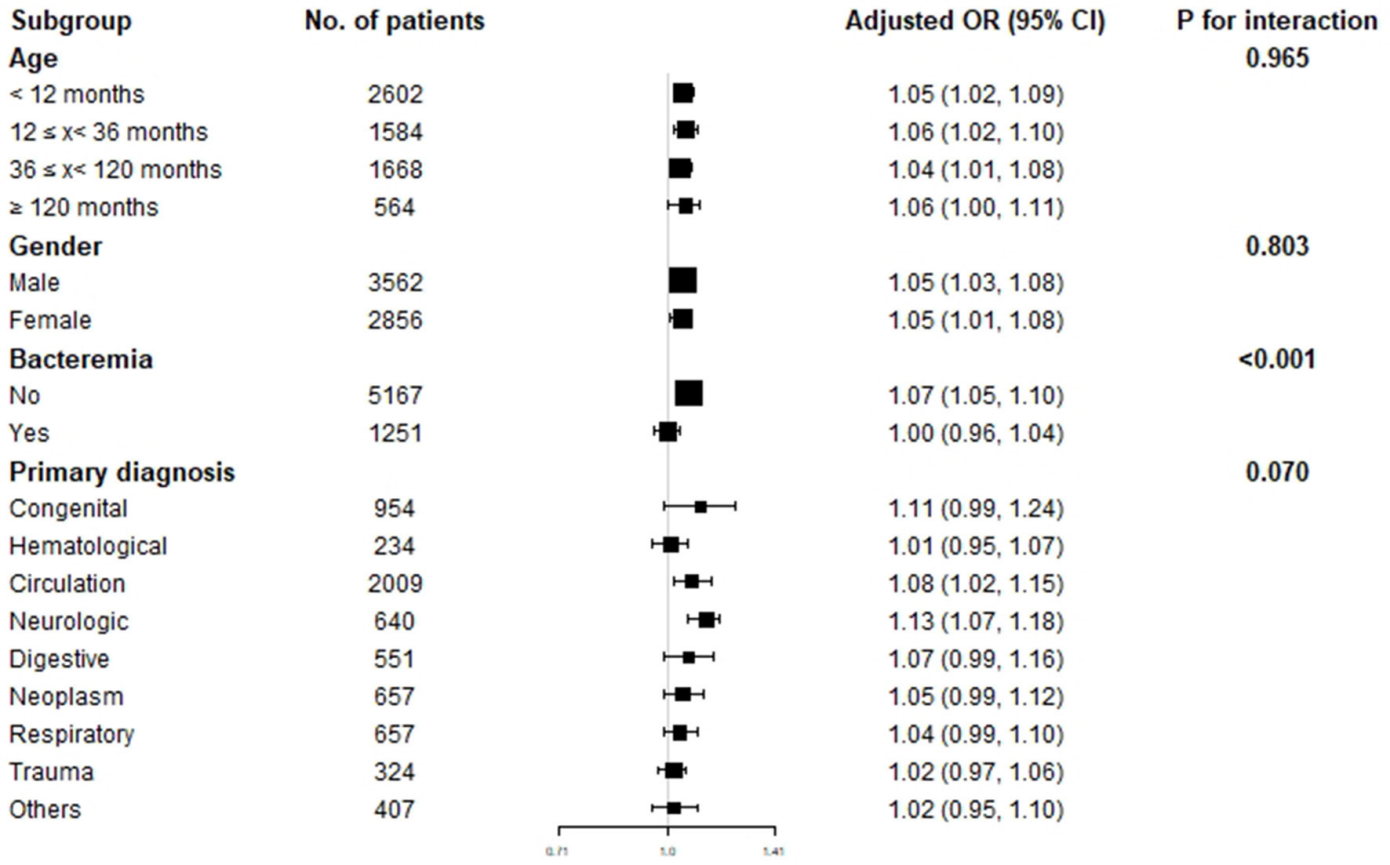
Subgroup analysis of the association between D-dimer and in-hospital mortality.

To explore the effect of age, gender, bacteremia, and primary disease on the relationship between D-dimer, INR, and in-hospital mortality of critically ill pediatric patients, we performed subgroup analysis grouping by age, gender, bacteremia, and primary diagnosis (Table 4). The results indicated that D-dimer had a strong and stable association with in-hospital mortality in different age groups (*P* for interaction = 0.965) and gender groups (*P* for interaction = 0.803). However, INR was only associated with in-hospital mortality in patients aged between 12 and 36 months (OR 1.63, 95% CI 1.12–2.36, *P* for interaction = 0.463) stratified by age and female patients (OR 1.31, 95% CI 1.07–1.61, *P* for interaction = 0.419) stratified by gender. Furthermore, D-dimer was also associated with in-hospital mortality in patients without bacteremia (OR 1.07, 95% CI 1.05–1.10, *P* for interaction <0.001) and patients with cardiovascular or neurological disorders (cardiovascular: OR 1.08, 95% CI 1.02–1.15, neurological: OR 1.13, 95% CI 1.07–1.18, *P* for interaction = 0.070) (Figure 3).

**Table 4:** Subgroup analysis of the association between D-dimer, INR and in-hospital mortality.

	***N***	**D-dimer**	***P*** **for interaction**	**INR**	***P*** **for interaction**
Age			0.965		0.463
<12 months	2602	1.05 (1.02, 1.09)		1.19 (0.93, 1.53)	
12 ≤ x< 36 months	1584	1.06 (1.02, 1.10)		1.63 (1.12, 2.36)	
36 ≤ x< 120 months	1668	1.04 (1.01, 1.08)		1.12 (0.64, 1.94)	
≥120 months	564	1.06 (1.00, 1.11)		1.12 (0.67, 1.87)	
Gender			0.803		0.419
Male	3562	1.05 (1.03, 1.08)		1.15 (0.89, 1.49)	
Female	2856	1.05 (1.01, 1.08)		1.31 (1.07, 1.61)	
Bacteremia			<0.001		0.021
No	5167	1.07 (1.05, 1.10)		1.37 (1.15, 1.64)	
Yes	1251	1.00 (0.96, 1.04)		0.87 (0.59, 1.29)	
Primary diagnosis			0.070		0.087
Congenital	954	1.11 (0.99, 1.24)		1.17 (0.60, 2.27)	
Hematological	234	1.01 (0.95, 1.07)		0.83 (0.30, 2.31)	
Circulation	2009	1.08 (1.02, 1.15)		1.43 (0.92, 2.21)	
Neurologic	640	1.13 (1.07, 1.18)		0.96 (0.37, 2.47)	
Digestive	551	1.07 (0.99, 1.16)		2.74 (1.64, 4.58)	
Neoplasm	657	1.05 (0.99, 1.12)		2.82 (0.29, 27.05)	
Respiratory	657	1.04 (0.99, 1.10)		1.15 (0.69, 1.92)	
Trauma	324	1.02 (0.97, 1.06)		1.25 (0.60, 2.63)	
Others	407	1.02 (0.95, 1.10)		0.99 (0.65, 1.51)	

Adjusted for age, sex, bacteremia, vasopressors use, WBC, hemoglobin, ALT, albumin, creatinine, lactate, CRP and principal diagnosis on ICU admission.

## Discussion

The prediction of prognosis for critically ill children in PICU is of great significance in clinical practice due to its relevance with therapeutic decisions. However, the diseases treated in PICU are complex and diverse, involving multiple systems and organs with different pathogenesis, thus bringing a great challenge for clinicians to predict the prognosis of unselected critically ill children.

In the present study, we found the coagulant function of non-survivors in critically ill children was significantly poorer than the survivor group. One study reported that in pediatric patients with septic shock, a worse clinical outcome can be more commonly seen in patients with coagulopathy than those without coagulopathy (5). Interestingly, our findings showed that D-dimer was most strongly associated with PICU in-hospital mortality among the complete coagulation indicators (PT, INR, APTT, and Fib) through calculation of AUC and adjusted OR. To our knowledge, we are the first to analyze the association of coagulation indicators and prognosis of pediatric critical illness in such a large sample size, and found D-dimer was a powerful predictive marker. Similarly, Yuhuko Ichkawa et al. (10) reported that an increased level of D-dimer was found in many underlying diseases and can predict a poor outcome in critically ill middle-aged and elderly patients. Another study indicated that pediatric patients with septic shock also showed a higher level of D-dimer in the non-survivor group (5). A prospective emergency department cohort study reported that compared to patients with abnormal D-dimer levels, patients with normal D-dimer levels presented a lower risk of 30-day mortality (18). Furthermore, the increased D-dimer levels were associated with higher production of TNF-α, IL-6, and IL-8 (19, 20) and indicated the activation of coagulation and fibrinolysis to develop to disseminated intravascular coagulation (DIC), especially in sepsis (20). All the undesirable biological processes may significantly increase the incidence of multi-system organ failure (MOF) (19), leading to high in-hospital mortality of critically ill children.

Logistic regression showed that among the coagulation indicators, D-dimer was an independent risk factor for PICU mortality. In the quartile analysis, D-dimer levels in the third and the fourth quartile groups (0.46–1.51 and 1.51–56.86) were significantly related to the raised risks of in-hospital mortality of the patients. Since the normal reference range of D-dimer is <0.55 mg/L, part of the population with normal D-dimer level (0.46–0.55 mg/L) were also included in the third quartile group. Therefore, clinicians should note that the in-hospital mortality risk in this population with D-dimer value 0.46–0.55 was probably overestimated in our study. The AUC of D-dimer level to predict the outcome was higher than other coagulation indicators (PT, INR, APTT, and Fib), which suggested that D-dimer levels had the best performance in predicting in-hospital mortality of critical illness in children. In critically adult patients, the AUC of D-dimer was also significantly higher than PT-INR (10). The cut-off value of the D-dimer level in predicting the prognosis of critically ill children was 1.53 mg/L, which was consistent with clinical application, indicating that when the D-dimer level is higher than 1.53 mg/L, the risk of death will be greatly increased. However, the cut-off value of the D-dimer level was 4.2 mg/L for predicting the outcomes of critically ill middle-aged and elderly patients (10). The main reason responsible for the difference may be the significant difference in the population age in the two studies. They mainly included the middle-aged and elderly patients, while we focused on children. Our study revealed that the sensitivity and specificity of D-dimer for predicting in-hospital mortality of critically ill patients were 65 and 77%, respectively. Another study with a small sample (46 cases) found that among pediatric patients who were admitted for trauma, a threshold of above 10 times of the reference upper limit on the first day of admission had a sensitivity of 90% and specificity of 100% for predicting mortality (21). The possible causes of the difference in sensitivity and specificity between the two studies may be the different sample size and study population. Therefore, further studies should be done in more certain diseases and larger populations to verify our results.

Subgroup analysis indicated that the D-dimer level was strongly and stably associated with in-hospital mortality independent of age and gender, which can be convenient and widely used in clinical practice. Moreover, for critically ill children with cardiovascular or neurological disorders, D-dimer was also most strongly associated with in-hospital mortality. Similarly, previous studies also reported that a high D-dimer level is an important independent and sustained risk factor for outcome and mortality in patients with cardiovascular diseases such as coronary disease (22), and neurological disorders such as spontaneous intracerebral hemorrhage (ICH) (23). Interestingly, we found that D-dimer was not associated with in-hospital mortality in patients with bacteremia. It was inconsistent with the findings of a previous study, which indicated that D-dimer was a biomarker for early prediction of clinical outcomes in patients with severe invasive infections due to Streptococcus pneumoniae and Neisseria meningitidis (24). Although increased D-dimer levels were proven to be associated with poor prognosis in numerous studies, others revealed that the predictive value of D-dimer for clinical outcomes in sepsis patients may be modest or poor (25–27). Moreover, a recent study reported that sepsis patients without D-dimer increase (<500 ng/mL) had a much higher mortality than those with moderate and marked D-dimer increase (*P* = 0.0003) (28). Therefore, D-dimer seems to be an interesting and controversial biomarker in predicting clinical outcomes in different models of infection. In our opinion, these opposite findings are not due to the inconsistency of D-dimer as a prognostic biomarker, but because its specificity is differently associated with specific conditions and specific pathogens.

We also found that Fib was a protective factor for critical illness in children, indicating that the decreased Fib level (<1.48 g/L) was relative to the poor prognosis of critically ill children. This is consistent with a previous study for pediatric critical illness which recommended that the low level of Fib (<1.50 g/L) was linked to high mortality (29). Nonetheless, Bredbaca et al. (30) reported that patients with early signs of hypercoagulation, as evaluated by elevated fibrin levels, would experience more organ failure and have higher in-hospital mortality compared to those with normal fibrin levels in ICU patients. Because these patients may be at different stages of coagulation dysfunction and in the advanced stage, a large amount of fibrinogen is degraded, resulting in a decline in fibrinogen levels and elevation in fibrin levels. However, the AUC of Fib in our findings for predicting in-hospital mortality was significantly lower than other coagulation indicators, suggesting that Fib was not valuable as an independent predictor of critical illness.

This study has some limitations. First, although our study included large sample size, it was a retrospective observational design with potential selection bias and confounding bias. The values of coagulation indicators were unavailable for some patients upon PICU admission, who were hence excluded from our study, for which the selection bias was inevitable. Also, it is a single-center study, which may limit the generalization of our results to a larger extent. Finally, the present study only collected the first data of D-dimer after PICU admission, with a lack of baseline level and continuous fluctuation analysis. Further prospective and multi-center studies are required to improve the reliability and generalizability of our findings. An intervention study could also be done based on D-dimer levels to reduce PICU mortality and enhance early recovery.

In conclusion, this study demonstrates that poorer coagulant function can be founded in the non-survivors compared with the survivors, indicating that the coagulation indicators play a crucial role in the prognosis evaluation of critical illness in PICU. Most importantly, among the coagulation indicators, D-dimer is most strongly associated with in-hospital mortality of critically ill children. Considering that this is a retrospective single-center study, further prospective and multi-center studies with a wider range of pediatric critical patients would be needed to validate our findings.

## Data availability statement

The raw data supporting the conclusions of this article will be made available by the authors, without undue reservation.

## Ethics statement

The studies involving human participants were reviewed and approved by Institutional Review Board of the Children's Hospital, Zhejiang University School of Medicine (Hangzhou, China). Written informed consent from the participants' legal guardian/next of kin was not required to participate in this study in accordance with the national legislation and the institutional requirements.

## Author contributions

GW and XL contributed to the study conception and design, performed data analysis, and revised the manuscript. JL contributed to the conception and design of the study, and wrote the first draft. RX contributed to data collection. All authors have read and approved the final manuscript.

## Funding

This work was supported by grants from the National Natural Science Foundation of China (82171352), Natural Science Foundation of Shandong Province (ZR201808010011), and Clinical Research Project of Shandong University in 2021 (Key Special Project for Critical and Critical Diseases).

## Conflict of interest

The authors declare that the research was conducted in the absence of any commercial or financial relationships that could be construed as a potential conflict of interest.

## Publisher's note

All claims expressed in this article are solely those of the authors and do not necessarily represent those of their affiliated organizations, or those of the publisher, the editors and the reviewers. Any product that may be evaluated in this article, or claim that may be made by its manufacturer, is not guaranteed or endorsed by the publisher.

## Supplementary material

Supplementary Figure 1 Non-linear cubic spline curve of D-dimer **(A)**, INR **(B)**, PT **(C)**, APTT **(D)** and Fib **(E)** against in-hospital mortality. The solid line represents the fitted line of the association between coagulation parameter and estimated OR of in-hospital mortality risk. The dotted line represents the upper and lower 95% CIs.

## Abbreviations

PICU: pediatric intensive care unit
PIC: pediatric intensive care
SLE: systemic lupus erythematosus
PT: prothrombin time
INR: international normalized ratio
APTT: activated partial thromboplastin time
Fib: fibrinogen
ORs: odds ratios
CIs: confidence intervals
WBC: white blood cell
PLT: platelet
ALT: alanine transaminase
CRP: C reactive protein
ROC: receiver operating characteristic
AUC: area under the curve
DIC: disseminated intravascular coagulation
MOF: multi-system organ failure.
