# Supplementary figures and images for: Elevated plasma D-dimer levels are associated with the poor prognosis of critically ill children
Source: Front Pediatr. 2022 Sep 23;10:1001893. doi: 10.3389/fped.2022.1001893 (PMC9537732; doi:10.3389/fped.2022.1001893)

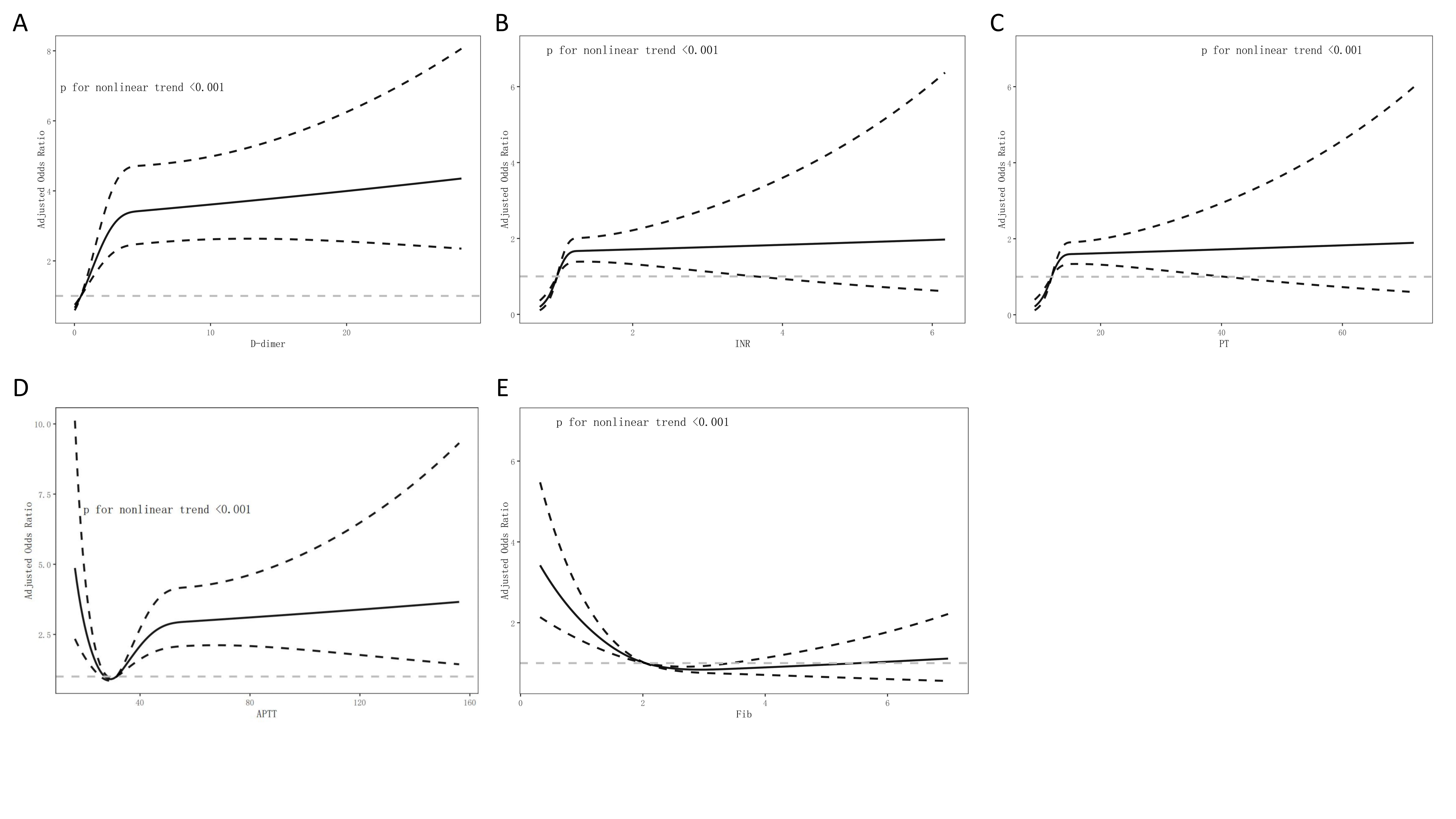

Supplement: Supplementary Figure 1 — Non-linear cubic spline curve of D-dimer (A), INR (B), PT (C), APTT (D) and Fib (E) against in-hospital mortality. The solid line represents the fitted line of the association between coagulation parameter and estimated OR of in-hospital mortality risk. The dotted line represents the upper and lower 95% CIs. [file Image_1.JPEG]
